# Multifunctional Dyeing of Textiles with Turmeric (*Curcuma longa* L.) Extracts Using Natural Mordants

**DOI:** 10.3390/molecules31183278

**Published:** 2026-09-16

**Authors:** Rong Yang, Changran Li, Yulong Zheng, Chao Huang, Ruyu Yao, Jianqin Li, Angkhana Inta, Lixin Yang

**Affiliations:** 1 Key Laboratory of Economic Plants and Biotechnology, Kunming Institute of Botany, Chinese Academy of Sciences, Kunming 650201, China; yangrong@mail.kib.ac.cn (R.Y.);; 2 Department of Biology, Faculty of Science, Chiang Mai University, 239 Huay Kaew Road, Chiang Mai 50200, Thailand; 3 Yunnan International Joint Laboratory of Southeast Asia Biodiversity Conservation, Xishuangbanna 666303, China; 4 School of Ethnic Medicine, Yunnan Minzu University, Kunming 650201, China; 5 School of Economics and Management, Southwest Forestry University, Kunming 650224, China; 6 Yunnan International Joint Laboratory of Health Plant Resources Development & Yunnan Key Laboratory for Wild Plant Resources, Kunming Institute of Botany, Chinese Academy of Sciences, Kunming 650201, China

**Keywords:** *Curcuma longa* L., natural mordants, technology optimization, color properties, multifunctional textile

## Abstract

Driven by concerns over environmental and human well-being, plant-based dyes have gained renewed attention as bio-based alternatives in textile coloration. Turmeric (the root of *Curcuma longa* L.) is a classical plant dye used extensively worldwide; however, it has relatively poor color fastness and limited availability, and its application in textiles requires optimization of dyeing parameters. In this study, the pigment composition of turmeric extracts was characterized by ultraviolet–visible (UV-Vis) spectroscopy, the fabrics were detected via SEM, and extraction and dyeing conditions were optimized through an orthogonal experimental design. To evaluate the impact of mordant type on the functional performance of turmeric-dyed fabrics, we compared natural and metallic mordants. This comparison was based on a comprehensive set of properties, including color depth (*K/S* values), chromatic parameters, color fastness, anti-ultraviolet, antioxidant capacity, and antimicrobial activity. The primary pigment component of turmeric extracts was curcumin, whose extraction was optimal at a material-to-liquor ratio of 1:8, using an 80% ethanol solution heated to 80 °C for 140 min. The ideal dyeing cotton conditions were pH 8, 70 °C, for 100 min. The application of natural mordants improved the color performances, UV protection, and antimicrobial performances of dyed samples but resulted in slightly inferior antioxidant activity compared to metallic mordants. The color fastness of dyed samples under mordants rose from moderate to excellent, apart from washing. This research highlights that turmeric-dyed natural fabrics with natural mordants could be a less hazardous option for eco-friendly textile production, providing both practical dyeing and functional properties.

## 1. Introduction

There is growing interest in reducing environmental pollution and generating more bio-based products using natural chemicals and optimized methods to enhance sustainability and reduce the chemical industry’s environmental impact [1]. Eco-friendly approaches are especially needed in textile manufacturing, which represents 75% of the global dye market and uses about 10,000 tons in the dyeing and printing process [2]. Each year around 280,000 tons of textile dyes are discharged into the environment through wastewater, posing major threats to the environment as well as animal and human health [2]. The extent of water pollution in the textile industry has raised concerns and prompted efforts to address the clean-water crisis by adopting natural dyes that are environmentally friendly [2,3].

Natural dyes originate from a variety of sources, including animals, plants, fungi, prokaryotes, and minerals. Among these, plant-derived dyes are the most commonly utilized due to their abundance, diversity, and ease of extraction [4]. Pigments may be isolated from various plant organs—including roots, stems, leaves, flowers, seeds, and fruits—each contributing distinct color characteristics and chemical compositions [5]. These pigments encompass a wide spectrum of naturally occurring bioactive molecules, including essential oils, flavonoids, terpenoids, and phenolic acids, which play crucial roles in plant coloration [6,7]. These bioactive molecules are multifunctional, exhibiting properties including antibacterial activity, antioxidant activity, and protection against ultraviolet (UV) light, which has driven their application in the health, medical, and pharmacological fields [8,9].

In addition, dyed fabrics made of natural fibers are vulnerable to damage from UV light, which produces free radicals [10]. To address these problems and improve natural fibers’ properties, recent research has focused on applying a range of functional finishes, in addition to dye, using chemicals extracted from plants [11]. Bioactive fabrics produced with plant-based dyes are emerging as an exciting trend, with potential applications extending to the biomedical field [12,13].

Although plant-based dyes can make textile dyeing more eco-friendly, such dyes also have downsides that limit their application and development [14]. The main drawbacks of plant-based dyes are their low color fastness (resistance to fading) and limited color range. In addition, standardized coloring has not been established; the use of plant-based dyes is costly, they are extracted from limited resources, and they have low color exhaustion (low transfer of the dye to the fabric), resulting in low utilization efficiency [15]. The color fastness of plant-based dyes can be improved by adding metallic mordants, which fix the color to the fabric by reacting with the pigment molecules and fibers to form complexes [16]. However, the amount of mordants participating in the reactions is limited, and the release of unspent mordants into wastewater can cause environmental problems and raise health issues [17,18]. Therefore, it is desirable to develop bio-based natural mordants as an alternative to metallic mordants [19,20].

Plant-based dyes can offer different colors for dyeing fabrics; in particular, many plants produce yellow dyes [21]. Turmeric (the root of *Curcuma longa* L.), a traditional source of yellow dye, has a wide range of applications worldwide [22]. It is a perennial plant in the Zingiberaceae family that grows mainly in India and China [23]. The main active pigment in turmeric roots is curcumin, which also has medicinal benefits, including anti-inflammatory, antioxidant, antitumor, and antifungal properties [24]. Curcumin on textiles is quantified by three methods: extraction followed by UV-Vis spectrophotometry, reflectance spectroscopy, and high-performance liquid chromatography (HPLC) [14]. Furthermore, the quantitative adsorption of curcumin on cotton fabric can be determined through kinetic model fitting [25]. Fabric dyeing with turmeric mainly involves either the addition of a metallic mordant [21], which confers functional finishing properties (antibacterial, antioxidant, and anti-ultraviolet activity) to the dyed fabric [12,26], or pretreatment of the fabric [27]. Dyeing with turmeric can also be accomplished via ultrasonic dyeing [28], microwave dyeing [29], or dye modification [30]. At present, research concentrated on the usage of natural mordants in turmeric-based dyeing remains limited. This is particularly evident for specific natural mordants, including soybean [*Glycine max* (L.) Merr.] seed, pomegranate (*Punica granatum*) peel, and plant ash (from *Pinus yunnanensis* Franch.), alongside mineral salts, rare earths, gum rosin and tartaric acid, the fruit of *Chaenomeles speciosa* (Sweet) Nakai, and chitosan, in dyeing fabrics with turmeric. Each natural mordant contains specific active compounds. Soybean seed: phenolic acids and flavonoids. Pomegranate peel: hydrolyzable tannins (punicalagin and ellagic acid). Plant ash: alkali carbonates and potassium salts. Mineral salts: metal ions (Al^3+^, Fe^2+^, and Cu^2+^) forming coordination complexes. Rare earths: high-coordination lanthanide ions (Ce^3+^ and La^3+^). Gum rosin: resin acids (abietic type). Tartaric acid: chelating organic acid. Chaenomeles speciosa fruits: triterpenes and flavonoids. Chitosan: cationic amino-rich biopolymer. These compounds enhance dye uptake, color fastness, and functional properties [11,31,32,33].

Although turmeric-based textile dyeing and functional modification have been widely reported, few studies have systematically compared natural and metallic mordants under identical experimental conditions, limiting the further development of sustainable turmeric dyeing systems. This work hypothesized that optimized natural mordants could confer dyed cotton fabrics with comparable or superior color performance and multifunctional properties relative to conventional metallic mordants, while offering improved environmental compatibility. Herein, orthogonal experiments were employed to synchronously optimize turmeric extraction and cotton dyeing processes. A total of nine natural and nine metallic mordants were systematically compared in terms of color depth, chromatic parameters, color fastness, UV resistance, antioxidant activity, and antibacterial performance, with further evaluation of the washing durability of the resulting multifunctional properties. This study clarifies the discrepancies between natural and metallic mordant systems and provides a feasible low-toxic and multifunctional strategy for eco-friendly turmeric textile coloration.

## 2. Results and Discussion

### 2.1. Spectroscopic Characterization Using UV-Vis

**Figure 1:**
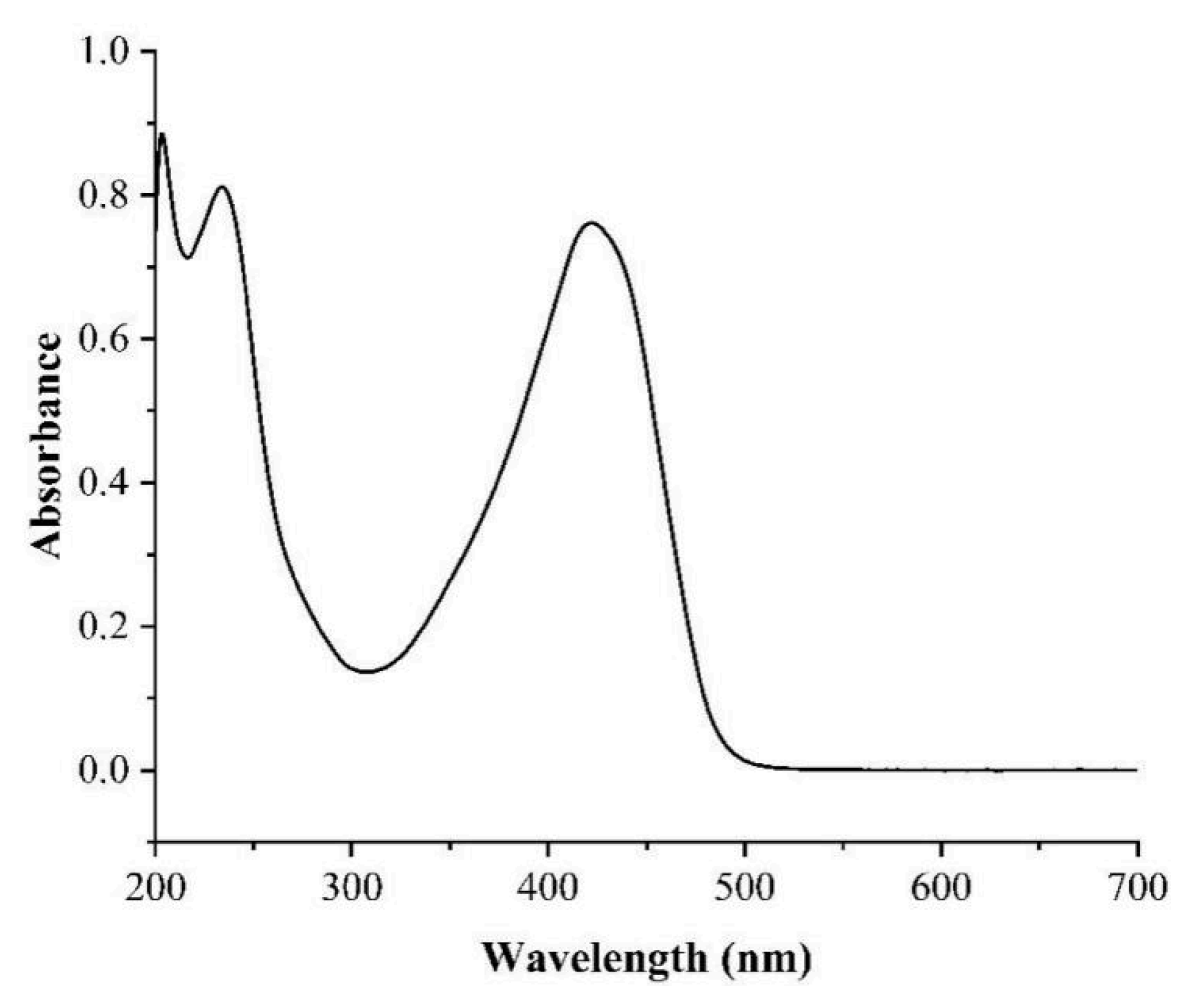
Absorption spectrum of the ethanol extract from turmeric roots.

Pigments were extracted from turmeric roots using ethanol under thermal conditions. As shown in Figure 1, the UV-Vis absorption spectrum of the extract exhibited two distinct maxima at 234 nm and 422 nm characteristic of curcuminoids [21]. The molecular structure of curcumin features a central β-diketone moiety that stabilizes the enol tautomer in a trans configuration [34]. The strong absorption of the turmeric root extract in the yellow region of the visible spectrum accounts for its vivid colorimetric properties.

**Figure 2:**
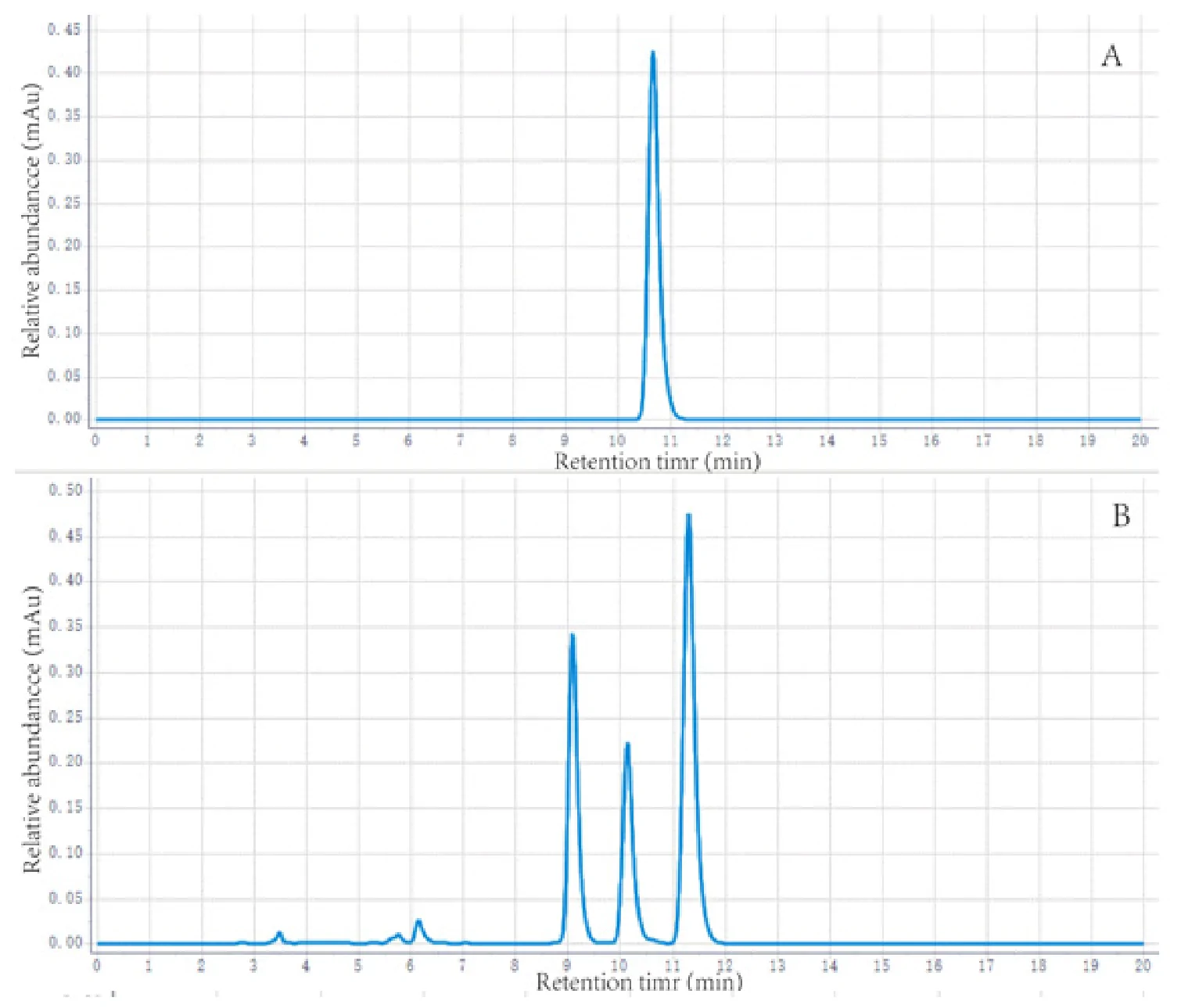
HPLC chromatogram ((**A**) curcumin standard; (**B**) sample).

As can be seen in Figure 2, comparing the HPLC chromatograms of the standard curcumin sample with those of turmeric, it was found that both samples contained curcumin.

### 2.2. Refining Extraction Conditions of Turmeric Roots

To systematically identify the optimal conditions for curcumin extraction from turmeric roots, an orthogonal array design was implemented, comprising a total of nine distinct experimental treatments. Table 1 compiles the absorbance values for the dye solutions derived from concentrated root extracts. Range analysis was employed, and the R value, which represents the range for each factor, was employed to assess the relative impact of individual parameters within the experimental design, and the four evaluated factors were ranked according to their influence on the measured absorbance. The level corresponding to the highest K value for each factor was then selected as the optimal condition.

We conducted a range analysis to calculate the Kij value as the summation of the absorption values for each of the four factors j (j = A, B, C, D) examined at three distinct levels, denoted as i = 1, 2, 3 in Table 1. And kij denotes the average of the summed absorbance values, and for each factor (j), its range value (Rj) was calculated as the range spanning the maximum and minimum values among the corresponding level averages (kij). In orthogonal experimental analysis, the magnitude of the Rj value reflects the relative influence of a factor, with a higher value denoting greater significance. The key parameters, kij and Rj, were computed in Table 1. For example,

k1A =(Σ(Absorption)1A)/3=(0.848+0.719+1.880)/3=1.149;

k2A =(Σ(Absorption)2A)/3=(0.578+0.899+0.547)/3=0.675;

k3A =(Σ(Absorption)3A)/3=(0.466+0.358+0.546)/3=0.457;

RA = max(kiA)−min(kiA) = k2A−k3A = 1.149−0.457= 0.692.

Based on the values shown in Table 1, we ordered the effect degree of the four factors on absorption in the order A > C > D > B from most important to least important. Thus, the material-to-liquor ratio (factor A) exerted the most significant effect on the absorbance of extracts derived from turmeric roots, followed by the temperature during extraction (factor C), the ethanol concentration (factor D) and the time of extraction (factor B). On the strength of this orthogonal design analysis, the optimal extraction condition of curcumin from turmeric roots is defined by A_1_C_3_D_3_B_3_, with a material-to-liquor ratio of 1:8 and a duration of extraction of 140 min at a temperature of 80 °C in 80% ethanol.

### 2.3. Optimization of Dyeing Conditions with Optimal Turmeric Root Extract

We dyed cotton fabrics using the optimal turmeric root extract identified above, varying the temperature, dyeing duration, and pH of the dye liquor. To evaluate the dyeing quality of the resulting fabrics, dyed samples were measured to detect the color depth, and a range analysis based on the orthogonal array design was performed, as shown in Table 2.

The dyeing behavior of cotton fabrics was primarily influenced by pH, followed by temperature and dyeing time. Therefore, pH was determined to be the key parameter influencing the dyeing performance of cotton fabric with ethanolic turmeric extracts, whereas dyeing time in cotton exerted comparatively minor effects. According to the orthogonal analysis, the optimal dyeing parameters were identified as G_2_E_1_F_3_ for cotton fabrics (operating at pH 8.0, 70 °C, for 100 min).

### 2.4. Impact of Mordanting Treatment on the Colorimetric Performance of Dyed Fabric

**Figure 3:**
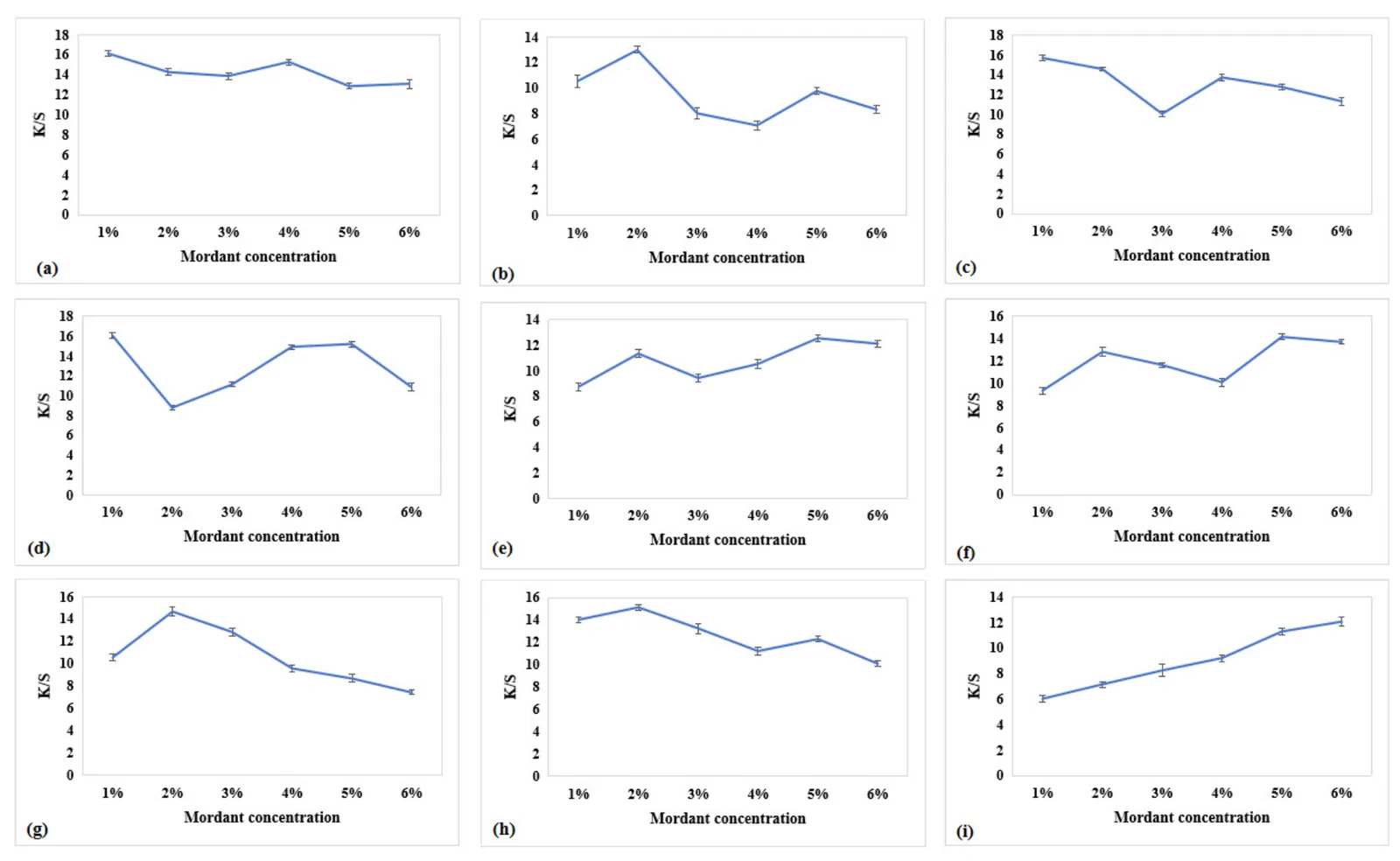
The influence of mordant concentration on the color intensity of turmeric-dyed cotton fabrics: (**a**) pomegranate peel; (**b**) *C. speciosa* fruits; (**c**) mineral salts; (**d**) tartaric acid; (**e**) plant ash; (**f**) rare earth; (**g**) gum rosin; (**h**) chitosan; (**i**) soybean seed.

The optimal mordanting concentrations for the nine natural mordants were screened via single-factor experiments. According to Figure 3, the optimum concentrations for rare earth, pomegranate peel, *C. speciosa* fruits, mineral salts, tartaric acid, plant ash, chitosan, gum rosin and soybean seed are 5%, 1%, 2%, 1%, 1%, 5%, 2%, 2% and 6%, in sequence.

Having established the ideal dyeing parameters for natural fabrics above, we turned our attention to the importance of mordants for the enhancement of color performance. To evaluate the effect of mordanting, the dyed cotton fabric was treated with two distinct sets of mordants: one comprising nine natural agents and the other nine metallic compounds. To quantitatively evaluate the effects of various mordants, the color strength (*K/S* values) and CIEL*a*b* coordinates for cotton fabric were determined after mordanting treatment. The corresponding results are presented in Table 3. The application of metallic mordants produced a diverse color palette ranging from yellow to dark brown, including light brown and greenish tones. In contrast, natural mordants generated predominantly yellow-based hues, encompassing light yellow, yellow-green, and a series of gray shades.

The definitive influence of mordant type on fabric color was quantified by measurable differences in the CIEL*a*b* coordinates, comprising lightness (L), the red–green axis (a), the yellow–blue axis (b), and the derived color difference metric (∆*E*). The efficacy of various mordants in enhancing the color strength of turmeric-dyed cotton fabrics was subsequently evaluated, with performance ranking in the following order: pomegranate peel > tartaric acid > mineral salts > chitosan > gum rosin > rare earth > *C. speciosa* fruits > plant ash > soybean seed > FeSO_4_ > AgNO_3_ > CuSO_4_ > CaCl_2_ > ZnSO_4_ > SnCl_2_ > KCl > KAl(SO_4_)_2_ > MgCl_2_ > no mordant. Natural mordants demonstrated a notable efficacy in enhancing color strength, with the treated fabrics exhibiting markedly higher *K/S* values compared to unmordanted counterparts (Table 3). Specifically, the color strength of dyed fabrics was highest when they were mordanted using pomegranate peel (16.26 for cotton) containing tannins; intermolecular h bonds between the OH groups of tannins and alkaloids improve the color intensity of dyed fabrics [35,36]. As *K/S* values represent color strength, an important indicator of color properties [37], it could be concluded that treatment with natural mordants distinctly improves the color performance of the dyed samples.

### 2.5. Ultraviolet Protection Performance of Dyed Fabrics

**Figure 4:**
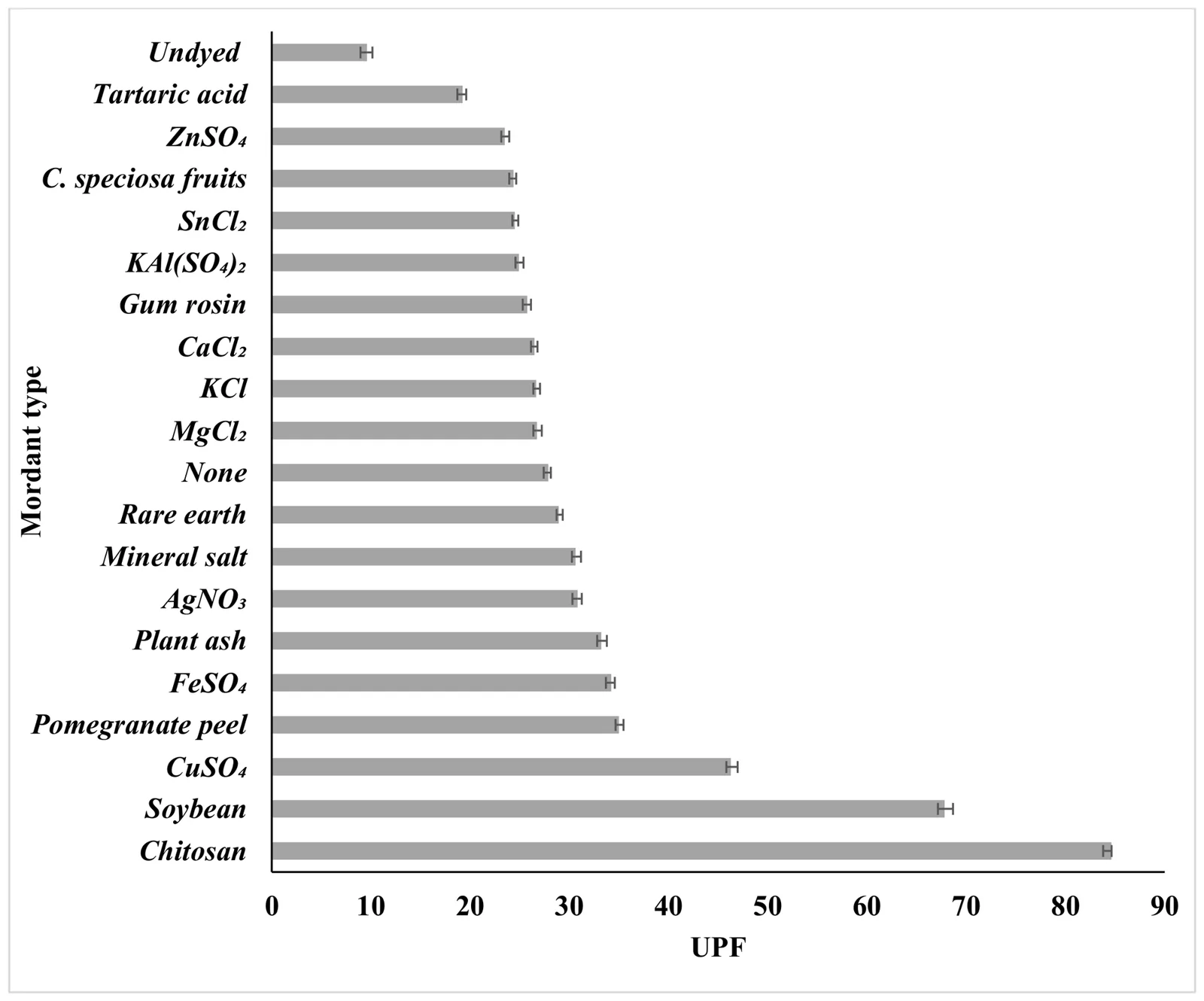
UPF values of turmeric-dyed cotton fabrics subjected to different mordants.

We measured the UV transmittance (%), UV protection (%), and UPF value of turmeric-dyed fabrics following treatment under different mordants (Table 4 and Table 5, Figure 4). Undyed fabrics demonstrated limited ultraviolet protection, with cotton exhibiting high transmittance and low blocking capabilities across UV spectra. Specifically, cotton transmitted 10.77% of UV-A and 9.45% of UV-B radiation. Correspondingly, the UV-A protection rates were 89.23% for cotton, whereas UV-B protection measured 90.55%. The ultraviolet protection factor (UPF) values further confirmed this trend, registering at 9.6 for cotton, indicating that these undyed fabrics have very poor UV protection qualities. By contrast, fabrics dyed with turmeric alone or followed by treatment with mordants had higher UPF values, which attributed to reduced transmittance and enhanced protection across the UV spectrum.

We ordered the effects of the mordants on UPF values of turmeric-dyed cotton fabric as follows: chitosan > soybean seed > CuSO_4_ > pomegranate peel > FeSO_4_ > plant ash > AgNO_3_ > mineral salts > rare earth > no mordant > MgCl_2_ > KCl > CaCl_2_ > gum rosin > KAl(SO_4_)_2_ > SnCl_2_ > *C. speciosa* fruits> ZnSO_4_ > tartaric acid (Table 4 and Table 5, Figure 3). Treating turmeric-dyed cotton fabric with chitosan yielded the highest UPF value of all mordants, resulting in fabrics with excellent UV protection properties. With reference to GB/T 18830-2009 (Textiles—Evaluation for solar ultraviolet radiation protective properties) [38], a fabric with a UPF value of 40 or above and a UV-A transmittance value of less than 5% can be considered as a UV-protecting textile. Therefore, turmeric-dyed cotton fabrics mordanted with CuSO_4_, soybean seed, or chitosan would be suitable for making functional textiles to defend wearers from ultraviolet rays.

The ultraviolet protective capability of dyed samples is governed by the chemical interplay between the applied dye, the mordanting agent, and the underlying fiber substrate [39,40]. Due to its cationic nature, chitosan can improve the dye absorption of cotton fabrics, enhancing their UV resistance [41]. The mechanism by which chitosan enhances the UV resistance of turmeric-dyed cotton fabrics includes the following: chitosan combines with cellulose of cotton fibers through hydrogen bonds and ionic bonds, forming a cationic polysaccharide film, providing more anchoring sites for curcumin and enhancing its uniformity and fastness of fixation [42]; the chitosan–curcumin composite coating complements the absorption of UV-A and UV-B bands, achieving broad-spectrum UV protection [43]; at the same time, chitosan forms a continuous and uniform covering layer on the fiber surface, enhancing the physical barrier effect and further improving the UV resistance [42].

**Figure 5:**
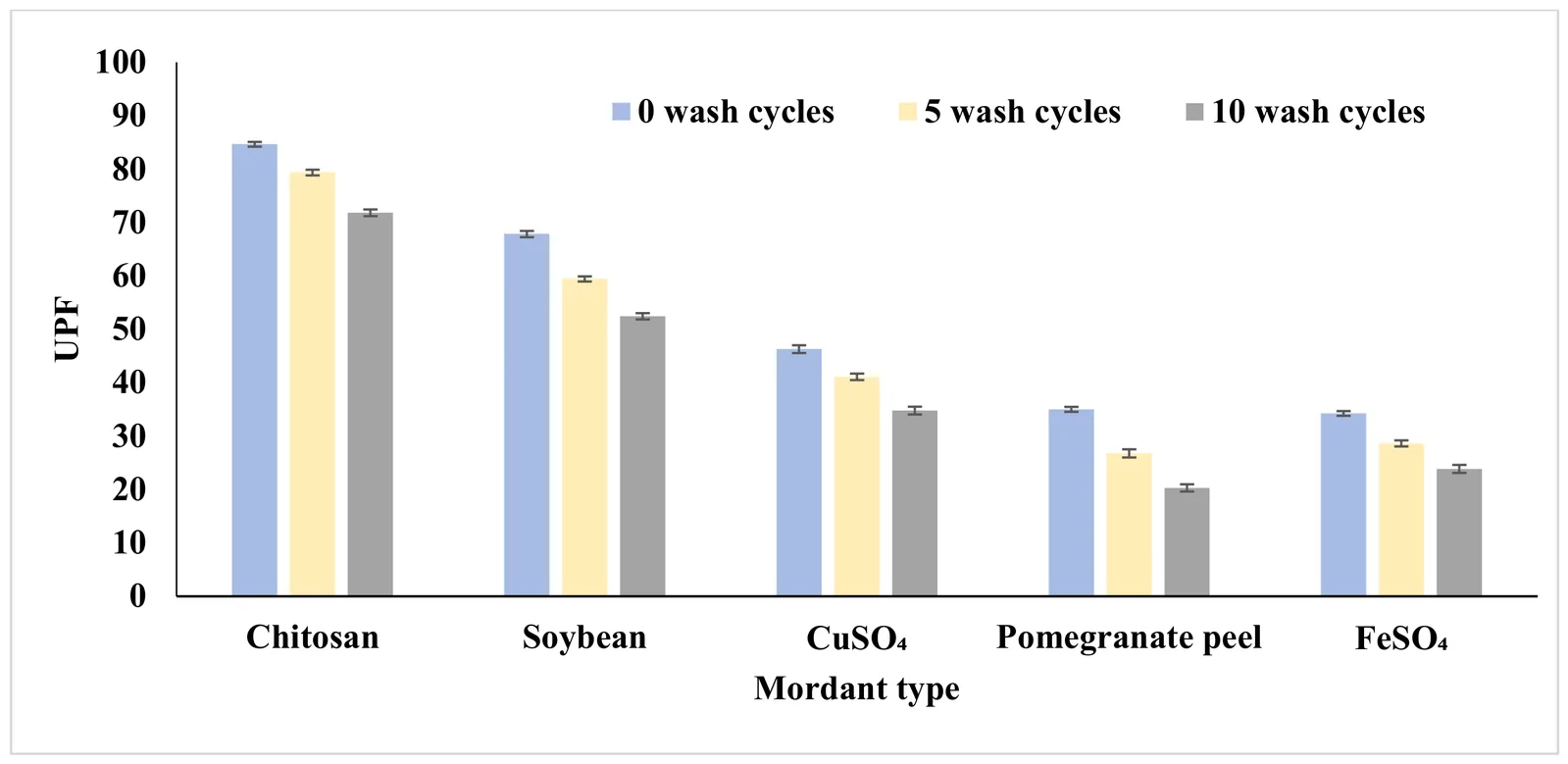
Stability assessments of UV resistance of turmeric-dyed cotton fabrics after repeated washing.

The top five mordants with superior ultraviolet (UV) resistance were selected to evaluate their washing fastness stability, and the corresponding results are presented in Figure 5. After 10 consecutive washing cycles, the fabric mordanted with soybean seeds exhibited the most pronounced deterioration in UV resistance, followed by the pomegranate-peel-mordanted sample. In contrast, the CuSO_4_-mordanted sample displayed the slightest variation in its UV protection performance. Even after 10 washing cycles, the chitosan-mordanted sample still possessed the optimal UV shielding capacity, with a UPF value reaching 71.82. Excluding the samples treated with pomegranate peel and FeSO_4_, all remaining specimens maintained UPF values higher than 40 following repeated washing, verifying their great potential for manufacturing UV-protective textiles.

### 2.6. Antioxidant Activity of Dyed Samples

**Figure 6:**
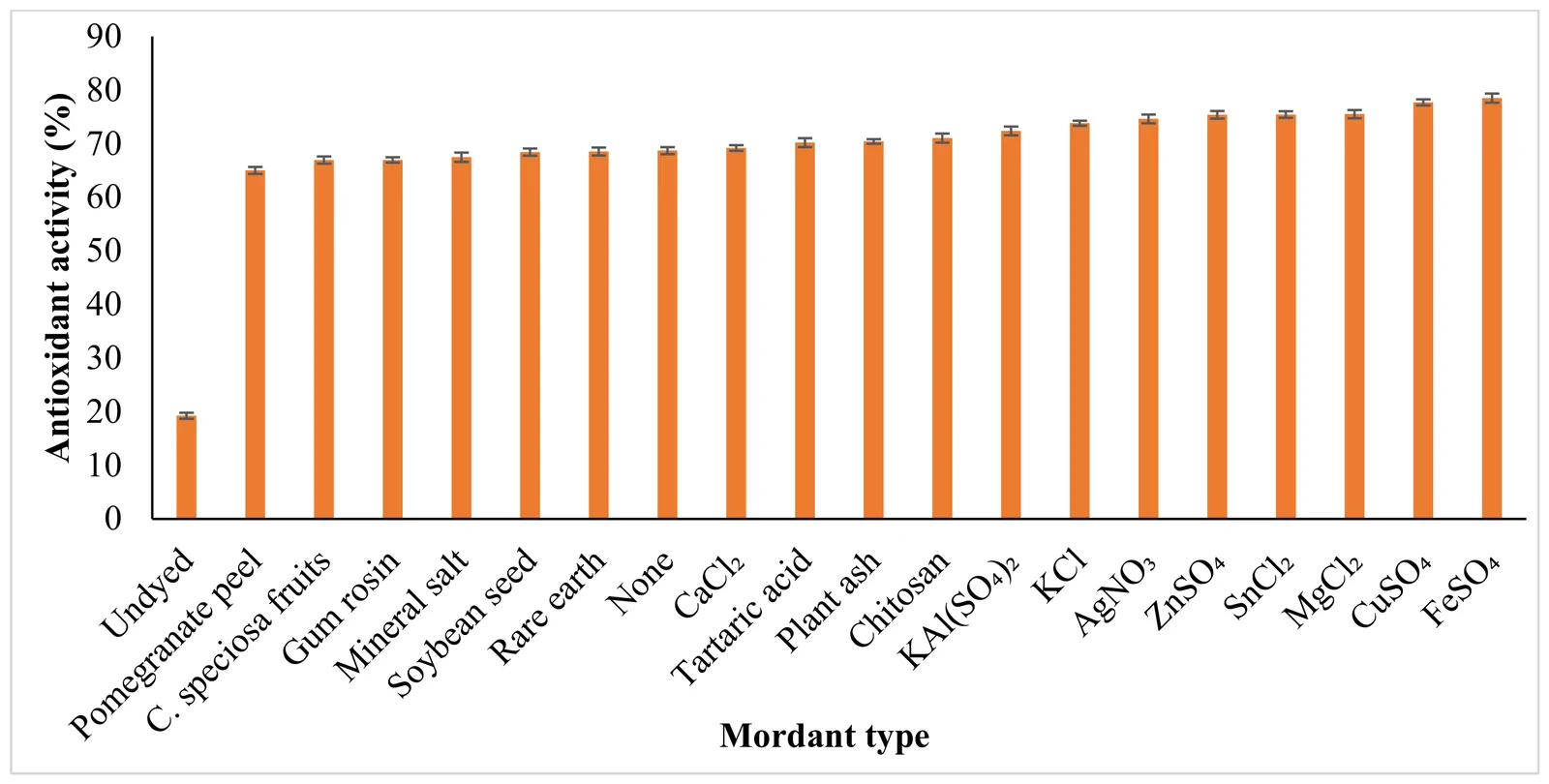
Antioxidant activity properties of turmeric-dyed cotton fabric subjected to different mordants.

Turmeric extracts have free radical scavenging capability, due to the chain-breaking ability of the phenol group of curcumin, releasing hydrogen atoms and methylene groups [44,45]. And under the different conditions, the oxidation resistances of turmeric-dyed natural fabrics were assessed via the DPPH assay (Figure 6). The antioxidant activity value of undyed cotton was 19.25%, indicative of relatively low free radical scavenging ability. By contrast, all turmeric-dyed cotton fabrics exhibited higher antioxidant activity, regardless of mordant treatment. We ordered the role of mordants for the antioxidant activity of turmeric-dyed cotton fabrics as follows: FeSO_4_ > CuSO_4_ > MgCl_2_ > SnCl_2_ > ZnSO_4_ > AgNO_3_ > KCl > KAl(SO_4_)_2_ > chitosan > plant ash > tartaric acid > CaCl_2_ > no mordant > rare earth > soybean seed > mineral salts > gum rosin > *C. speciosa* fruits > pomegranate peel. Thus, turmeric-dyed fabrics treated with FeSO_4_ displayed the highest antioxidant activity (78.48% for cotton). We hypothesize that this effect might be related to synergistic effects of ferrous ions and pigments during dyeing [46,47]. The mechanism by which FeSO_4_ enhances the antioxidant properties of turmeric-dyed cotton fabrics is attributed to the multiple synergistic effects of curcumin’s phenolic hydroxyl radical scavenging, the redox synergy of Fe^2+^/Fe^3+^, the enhancement of dyeing by mordanting, and the deep color light shielding effect [48,49]. Notably, the antioxidant properties of turmeric-dyed fabrics not treated using mordants were in the same range as those of fabrics treated with mordants, which may reflect an interaction between cotton fibers, dye–natural mordant complexes, and small amounts of metal ions present in natural mordants, leading to the quenching of free hydroxyl groups [50,51].

**Figure 7:**
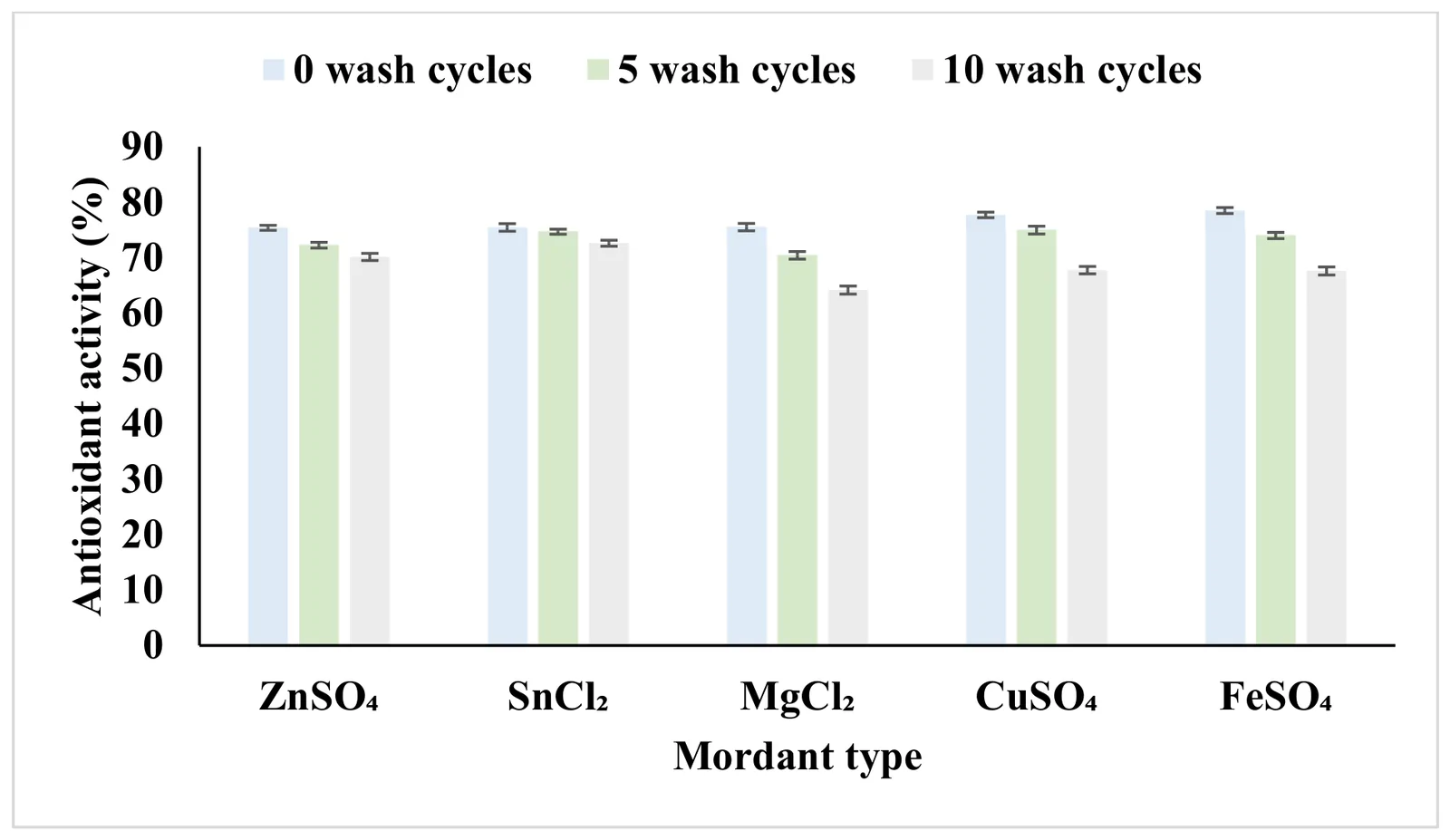
Stability assessments of antioxidant activity of turmeric-dyed cotton fabrics after repeated washing.

The top five mordants with superior antioxidant performance were selected to evaluate their washing fastness stability, and the corresponding results are presented in Figure 7. After 10 consecutive washing cycles, the fabric sample mordanted with MgCl_2_ exhibited the most significant reduction in antioxidant activity, followed by the FeSO_4_-mordanted specimen. In contrast, the stannous chloride SnCl_2_-mordanted sample showed the slightest variation in antioxidant properties, retaining 72.61% of its original antioxidant activity after 10 washing cycles. Notably, although the antioxidant activity of all five metal-mordanted samples decreased to varying degrees following repeated washing, their residual antioxidant activities remained above 60%.

### 2.7. Assessment of Antimicrobial Efficacy of Colored Samples

To assess the antibacterial efficacy of the turmeric-dyed textiles, the standardized tests against two common bacterial pathogens were conducted: the Gram-positive *S. aureus* and the Gram-negative *E. coli*, as presented in Table 6. Significant antibacterial effects were observed, which may be due to curcumin’s capacity to penetrate and disrupt the structural integrity of bacterial cell membranes, subsequently interfering with essential cellular proteins [52]. Mordant treatment substantially improved the antimicrobial efficacy of turmeric-dyed natural fabrics, demonstrating enhanced inhibitory effects against both *S. aureus* and *E. coli*. Notably, the use of the natural mordant derived from pomegranate peel produced the highest antibacterial activity (largest ZOI [zone of inhibition]), followed by the metallic mordants CuSO_4_ and SnCl_2_. The phenolic compounds in pomegranate peel (e.g., tannins, flavonoids, and ellagitannins) are capable of forming strong hydrogen bonds and hydrophobic interactions with cellulose fibers, as well as with bacterial cell walls. These interactions confer dual benefits by improving dye fixation and enhancing antibacterial efficacy, thereby inhibiting bacterial growth through membrane disruption and protein precipitation [53,54]. Meanwhile, a synergistic effect between pomegranate peel and turmeric was observed, enhancing the antibacterial efficacy. Tannins from pomegranate peel further stabilized curcumin on the fibers, resulting in a more durable antimicrobial finish [55]. Furthermore, the turmeric-dyed fabrics exhibited stronger antimicrobial effects against *S. aureus* than against *E. coli*, likely due to the distinct structural compositions of their cell walls. As a Gram-positive bacterium, *S. aureus* lacks an outer membrane, allowing the hydrophobic curcumin molecules to penetrate more readily and thereby improving the adhesion and release of active compounds from the fabrics [56]. While the inhibition-zone diameter provides a qualitative measure of antibacterial activity and can be adopted for preliminary screening, this assay has inherent limitations. For subsequent quantitative assessment of antibacterial performance, standard metrics, including bacterial reduction rates and viable colony counts of test fabrics, should be employed.

**Figure 8:**
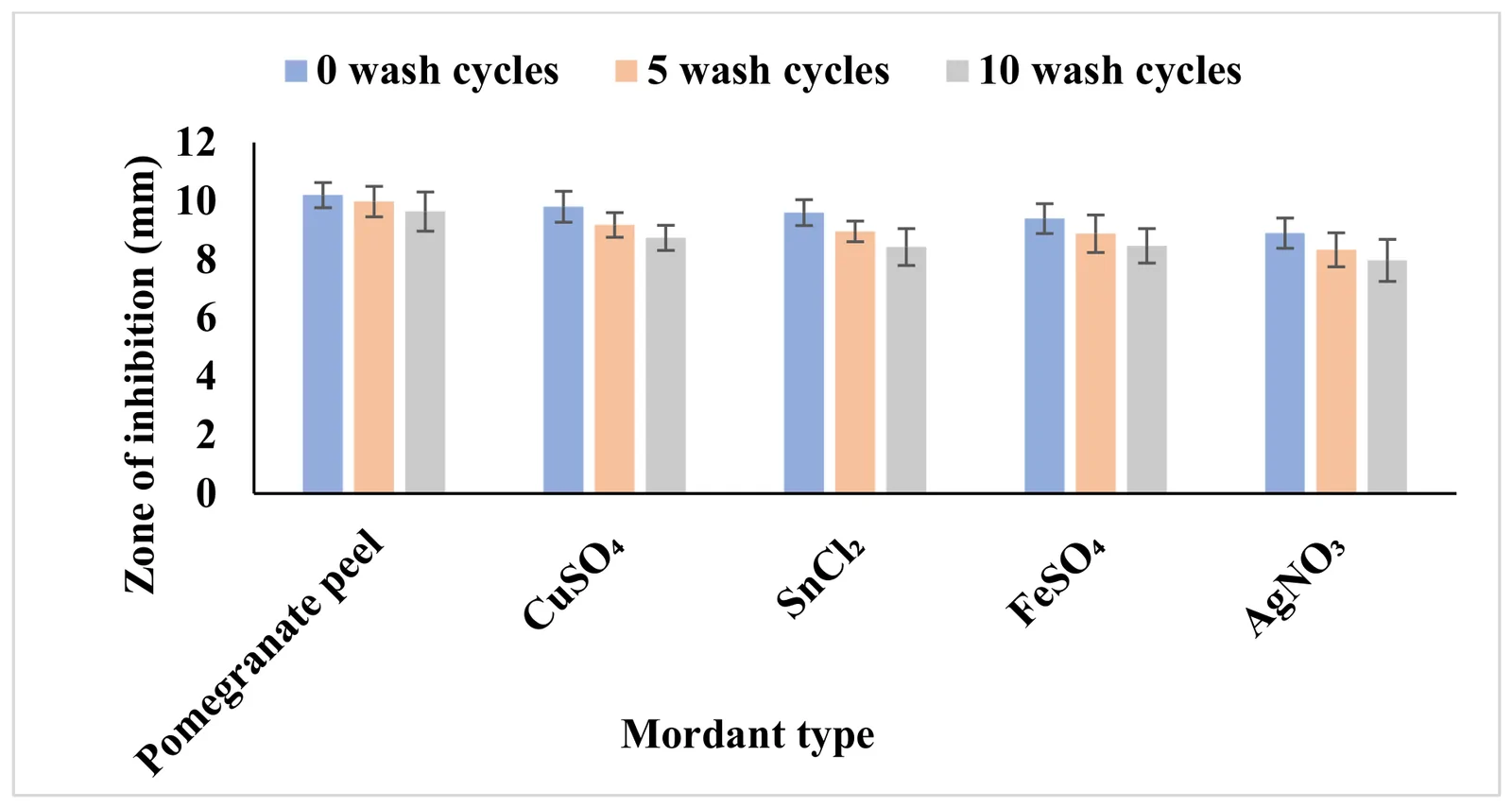
Stability assessments of antibacterial activity (*S. aureus*) of turmeric-dyed cotton fabrics after repeated washing.

**Figure 9:**
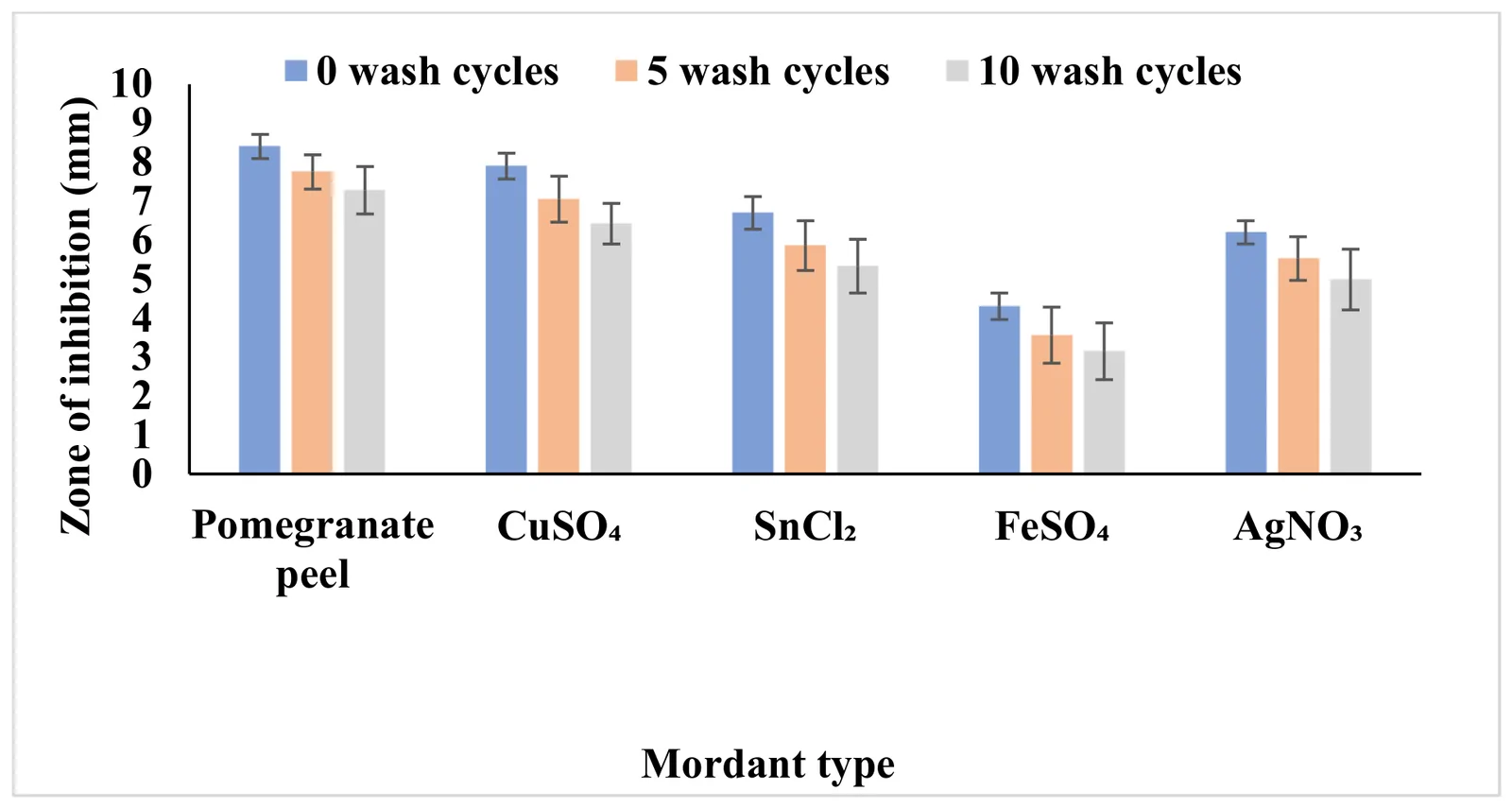
Stability assessments of antibacterial activity (*E. coli*) of turmeric-dyed cotton fabrics after repeated washing.

The top five mordants with superior antibacterial performance were selected to evaluate their washing fastness stability, and the corresponding results are presented in Figure 8 and Figure 9. After 10 consecutive washing cycles, the dyed samples exhibited a greater reduction in antibacterial activity against *E. coli* than against *S. aureus*, indicating better stability of the dyed fabrics against *S. aureus*. Furthermore, the dyed samples still displayed stronger antibacterial efficacy against *S. aureus* than against *E. coli* following repeated washing. Notably, the samples mordanted with CuSO_4_ and SnCl_2_ showed more pronounced declines in antibacterial activity. The pomegranate-peel-mordanted sample still retained the highest antibacterial activity, with inhibition zone diameters of 7.28 mm and 9.64 mm against *E. coli* and *S. aureus*, respectively, after 10 washing cycles. Except for the FeSO_4_-mordanted sample, which exhibited lower antibacterial activity against *E. coli* than the directly dyed control sample without mordanting after 10 washing cycles, all other specimens showed superior antibacterial performance compared to the control.

### 2.8. The Surface Morphology of Fabric Fibers

**Figure 10:**
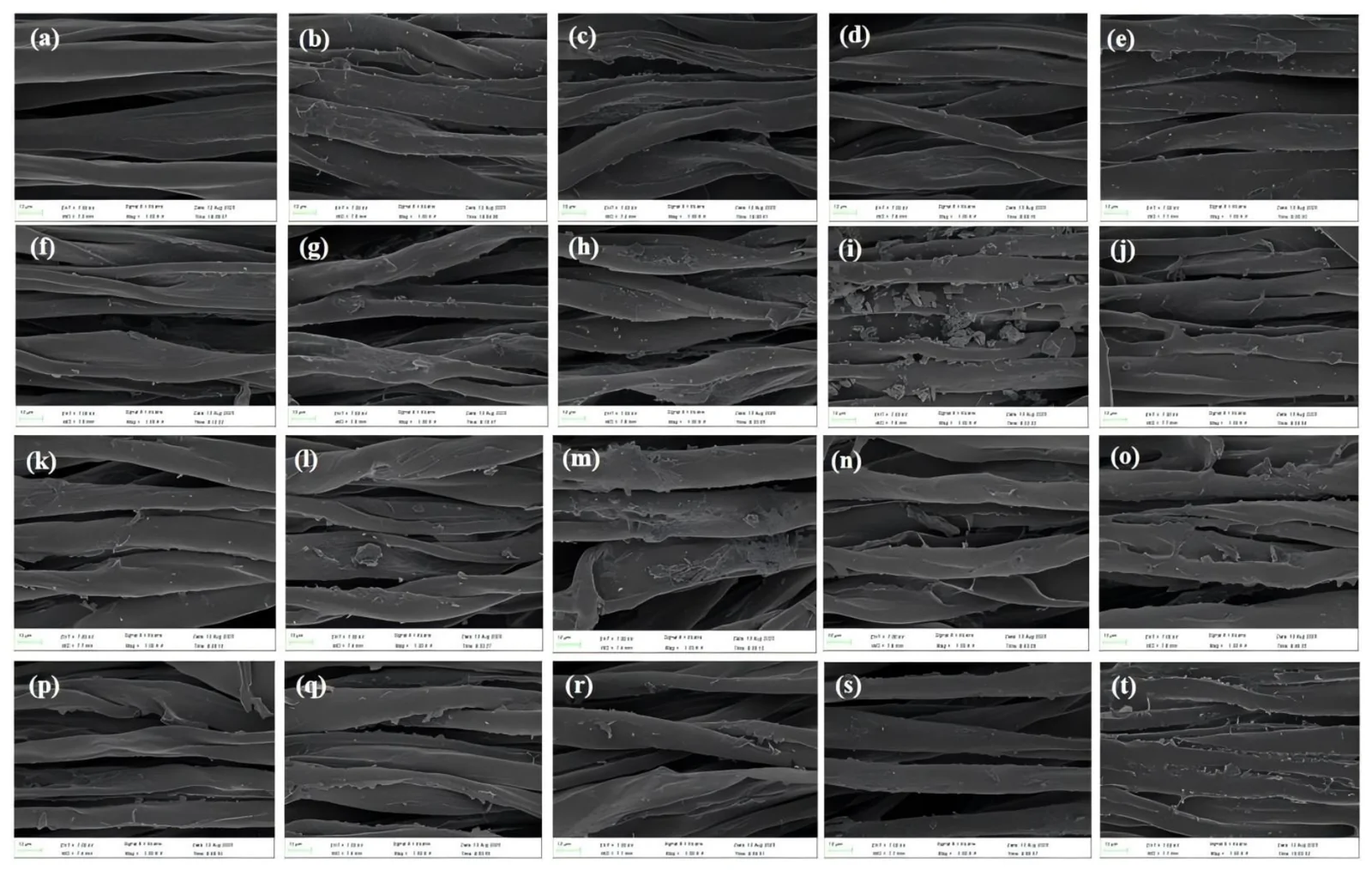
SEM images of undyed cotton fibers (**a**) and turmeric-dyed cotton fibers with different mordants ((**b**) sample without mordant, (**c**) sample with soybean seed, (**d**) sample with rare earth, (**e**) sample with chitosan, (**f**) sample with pomegranate peel, (**g**) sample with gum rosin, (**h**) sample with *C. speciosa* fruits, (**i**) sample with plant ash, (**j**) sample with mineral salts, (**k**) sample with tartaric acid, (**l**) sample with KAl(SO_4_)_2_, (**m**) sample with SnCl_2_, (**n**) sample with CuSO_4_, (**o**) sample with FeSO_4_, (**p**) sample with ZnSO_4_, (**q**) sample with KCl, (**r**) sample with MgCl_2_, (**s**) sample with CaCl_2_, (**t**) sample with AgNO_3_).

The surface morphologies of undyed, dyed, and mordanted dyed fabrics are presented in Figure 10. Scanning electron microscopy (SEM) revealed distinct differences between dyed and undyed fibers. As shown in Figure 10a, undyed cotton fibers exhibited smooth and even surfaces, indicating an unaltered state. In contrast, the fibers in Figure 10b displayed rough textures with particulate deposits on their surfaces under the no-mordant condition [57]. Furthermore, the application of mordants led to increased particle density and surface roughness in Figure 10c–t.

### 2.9. Color Fastness of Dyed Samples with Turmeric

The color fastness properties—including washing, rubbing, and perspiration resistance—of colored samples of all turmeric-dyed cotton fabrics were evaluated using a standardized 1–5 grading scheme (1: poor, 5: excellent). The corresponding results are summarized in Table 7. Mordant-assisted dyeing significantly improved color fastness properties, with treated fabrics demonstrating markedly better performance than their untreated counterparts. Specifically, non-mordanted fabrics showed limited resistance to perspiration-induced color change, with ratings of only 1–2, but reached a rating of 3–4 and a rating of 4–5 for dyed cotton fabric when mordanted with *C. speciosa* fruits. The color staining behavior of the dyed fabrics with mordants during washing and perspiration had a rating of at least 4 and sometimes as high as 5, depending on the mordant used. Notably, in perspiration fastness tests, the color change of the mordant-treated fabrics was lower than their color staining, indicating more favorable stain resistance than color preservation. The dyed fabrics exhibited low color change after washing, fabrics mordanted with ferrous sulfate achieving the highest fastness grades, with cotton reaching a grade of 3. We speculate that the pH sensitivity of curcumin in the turmeric extracts causes the low washing fastness of turmeric-dyed fabrics [58,59]. Furthermore, only the ∆*E* values of the fabrics dyed with CuSO_4_ and FeSO_4_ were lower than 2, indicating that the color differences caused by washing could only be noticed upon careful observation. The dyed fabrics mordanted prior to coloring displayed good to excellent rubbing fastness, with dry and wet ratings between 4 (good) and 5 (excellent), meeting textile industry standards.

## 3. Materials and Methods

### 3.1. Materials

Turmeric samples were collected in August 2023 from Dexing Village (29°19′38″ N, 95°17′27″ E), Beibeng Township, Medog County, Lingzhi City, in the Xizang Autonomous Region of China. The plant materials utilized in this study, including *C. speciosa* fruits, gum rosin, soybean seeds, pomegranate peel, and plant ash, were procured from a local bazaar situated in Xizhou Town, within the Dali Bai Autonomous Prefecture of China. Additionally, the mineral salts used in this experiment were sourced from Keko Town, located in Wulan County, Qinghai Province, China. Tartaric acid, chitosan, rare earth reagent, and nine metallic mordants (potassium aluminium sulfate, ferrous sulfate, stannous chloride, copper sulfate, silver nitrate, magnesium chloride, potassium chloride, calcium chloride, and zinc sulfate) were of laboratory reagent grade and purchased from Sinopharm Chemical Reagent Co., Ltd. (Shanghai, China). Anhydrous ethanol, sodium hydroxide (NaOH), and hydrochloric acid (HCl) were of analytical reagent grade and supplied by Sinopharm Chemical Reagent Co., Ltd. (Shanghai, China). All chemicals were used directly without further purification. Bleached cotton fabrics were bought from the Xingwei Ethnic Craft Factory, Taizhou, China. The test material was 100% plain-woven cotton fabric with an areal density of 135 g/m^2^ and a yarn count of 40 × 40 tex. The fabric was desized, scoured and bleached without additional finishing agents to avoid auxiliary interference. Specimens of 10 × 10 cm were prepared for dyeing and color fastness tests.

### 3.2. Extraction of Turmeric-Based Dye

Turmeric roots were washed with water and mashed into a pulp. The pigments were extracted using 60%, 70%, or 80% ethanol (all *v*/*v*) at a material-to-liquor ratio (*w*/*v*) ratio of 1:8, 1:10, or 1:12, at temperatures of 60 °C, 70 °C, or 80 °C, for 100 min, 120 min, or 140 min, based on an orthogonal experiment comprising nine distinct measurement conditions. Following cooling to room temperature, the pigment extracts were filtered, yielding the final dyeing solution.

### 3.3. Production of Natural and Metallic Mordants

Nine metallic mordant solutions were produced by dissolving each metal salt in distilled water at a concentration of 1% (*w*/*v*). Natural mordants are defined as bio-based or naturally sourced mordanting agents, and Table 8 presents the relevant information on the natural mordants used in the experiment. The natural mordants of soybean seeds were grouped into a slurry, and water was added to produce a 6% (*w*/*v*) solution in distilled water. The other natural mordants were prepared at the following concentrations in distilled water: 5% (rare earth elements), 2% (chitosan), and 1% (pomegranate peel, mineral salts, and tartaric acid) in distilled water. Gum rosin was first ground into a fine powder at ambient temperature using a mortar and pestle. This powder was subsequently dissolved in a 2:5:2 (*w*/*v*/*v*) ethanol-to-water mixture to prepare a 2% (*w*/*v*) mordanting solution. Dry *C. speciosa* fruits were thoroughly washed, sliced, and oven-dried. Subsequently, 2 g of the processed material was refluxed with 100 mL of distilled water for 30 min. The resulting mixture was filtered through qualitative filter paper to obtain the natural mordant extract. A 5% (*w*/*v*) plant ash solution was prepared by heating in distilled water for 30 min, followed by a 3 h sedimentation period. The resulting supernatant was filtered and collected for subsequent use as a natural mordant.

### 3.4. Ultraviolet–Visible Spectroscopy

UV–visible spectroscopy detection of turmeric extracts was implemented through scanning in the 190–600 nm wavelength range using a UV-6100PC spectrophotometer (Shanghai Yuanxi Instrument Co., Ltd., Shanghai, China) [60,61].

### 3.5. Orthogonal Experimental Optimization of Turmeric Dye Extraction and Dyeing Processes

To systematically optimize turmeric dye extraction and its application in textiles, we employed an orthogonal experimental design targeting cotton fabric. This study focused on four critical extraction parameters and three critical dyeing parameters to optimize the extraction and dyeing process. Within the orthogonal experimental framework, each extraction and dyeing parameter was tested at three distinct levels, designated as 1, 2, and 3. The specific values corresponding to these levels for each factor are detailed in Table 9 and Table 10.

### 3.6. Dyeing

Each turmeric extract obtained as described above was used to dye natural fabrics at a 1:30 fabric-to-dye bath ratio. The tested parameters included material-to-liquor ratio (the ratio of solid turmeric roots to ethanol solution: 1:8, 1:10, or 1:12), extraction temperature (60 °C, 70 °C, or 80 °C), dyeing time (80, 90, or 100 min), and ethanol concentration (60%, 70%, or 80%). Upon completion of the dyeing cycle, all fabric samples underwent a thorough rinsing with distilled water to remove unbound dye, followed by ambient-air-drying.

### 3.7. Mordanting Fixation Process

In this experiment, the post-mordanting process was carried out based on the previous field research and the relevant literature [7,62]. Dyeing was performed at temperatures ranging from 70 to 90 °C for 80–100 min under pH conditions of 5.0–9.0, using a constant liquor ratio of 1:30 (fabric weight to dye solution volume) to identify the ideal dyeing parameters. After dyeing, fabrics were transferred to a mordanting solution and incubated at 80 °C for 80 min. Ultimately, all samples were meticulously rinsed with distilled water to remove unfixed dye and subsequently air-dried under ambient temperature.

### 3.8. The Measurements of Color Parameters

Color parameters of dyed fabric specimens were acquired based on the CIE-Lab color space system. The CIE lightness (L*), red–green chromatic component (a*), and yellow–blue chromatic component (b*) were determined using a benchtop spectrophotometer (3NH TS600; Shenzhen ThreeNH Technology Co., Ltd., Shenzhen, China). All tests were performed under the D65 standard illuminant with a 10° standard observer angle, including specular reflection components, with a measuring aperture of 10 mm. Undyed cotton fabrics were employed as blank controls. For each sample, three different positions were randomly tested, and the average value was adopted for statistical analysis. The color strength (*K/S*) values of the dyed fabrics were calculated according to the Kubelka–Munk equation:

$$K/S\;=\;{(1-R)}^{2}/2R$$

Here, *R* corresponds to the reflectance at the wavelength of maximum absorption, while *K* and *S* denote the absorption and scattering coefficients of the dyed fabric, respectively.

### 3.9. Assessment of Color Fastness

All dyed fabric specimens underwent comprehensive color fastness testing following the Chinese Textile Testing Specifications, procedures that are consistent with the relevant ISO international standards. The color fastness of the dyed fabrics to washing, perspiration, and rubbing was evaluated following the corresponding Chinese National Standards: GB/T 3921-2008 [63], GB/T 3922-1995 [64], and GB/T 3920-2008 [65], respectively. Washing fastness (GB/T 3921-2008): Composite specimen (textile + adjacent fabric) in soap solution (liquor 50:1) rotates at 40 ± 2 r/min in a thermostated bath (40–95 °C) for 30–45 min. Rinse, dry, rate. Perspiration fastness (GB/T 3922-1995): Composite immersed in artificial sweat (pH 5.5 or 8.0, L-histidine + NaCl) for 30 min, then pressed at 12.5 kPa, 37 ± 2 °C for 4 h. Dry ≤ 60 °C, rate. Rubbing fastness (GB/T 3920-2008): Specimen clamped flat. Cotton rubbing cloth under 9 ± 0.2 N moves 10 cycles (dry or wet with 95–100% pick-up).

### 3.10. UV Protection Assessment

The ultraviolet (UV) protective performance of the fabrics was assessed in accordance with the Chinese National Standard GB/T 18830-2009. Key metrics—namely, the ultraviolet protection factor (UPF), transmittance across UV-A and UV-B bands, and the corresponding protection categories—were determined using a YG912E textile UV resistance tester (MEBON Instrument Technology Co., Ltd., Ningbo, China). Each sample underwent a minimum of three measurements, and the results are presented as mean values.

### 3.11. Antioxidant Properties Test

The radical-scavenging capacity of the treated fabrics and fibers was assessed using the DPPH assay. For antioxidant extraction, 0.5 g of cut dyed fabric was extracted with anhydrous ethanol at a solid–liquid ratio of 1:100 (g/mL). Ultrasonic-assisted extraction was conducted at 30 °C and 120 rpm for 60 min. A Trolox calibration curve was used for the quantitative determination of antioxidant activity. To guarantee detection accuracy, a reagent blank (ethanol + DPPH solution) and an untreated fabric extract blank were prepared for background correction. Moreover, the absorbance of extracts from mordant-only treated fabrics was measured and subtracted to eliminate color interference derived from mordant residues [66,67].

### 3.12. Antibacterial Activity Test

The antibacterial performance of dyed fabrics was qualitatively evaluated against *Staphylococcus aureus* (ATCC 6538, Gram-positive) and *Escherichia coli* (ATCC 8739, Gram-negative) via the agar diffusion method, in accordance with the national standard GB/T 20944.1–2007 [68]. Both bacterial strains were obtained from the China General Microbiological Culture Collection Center (CGMCC). The bacterial suspension was adjusted to a concentration of 1 × 10^6^ CFU/mL prior to inoculation. Each group was equipped with three parallel fabric specimens, and all tests were performed in triplicate. After inoculation, the plates were incubated at 37 °C for 24 h at a constant temperature in a humidity incubator. The antibacterial effect was qualitatively assessed by observing and measuring the diameter of the inhibition zone surrounding each fabric sample [69,70].

### 3.13. The Measurement of SEM

The surface morphologies of undyed and dyed cotton fibers were characterized via scanning electron microscopy (JEOL 6460LV; JEOL Ltd., Tokyo, Japan). Prior to observation, all fabric samples were dried and uniformly coated with a thin gold layer for 60 s to enhance surface conductivity. Microscopic imaging was performed at an accelerating voltage of 7.0 kV with a working distance of approximately 10 mm.

## 4. Conclusions

This study investigates a bio-based dyeing strategy that utilizes natural mordants to improve both the color quality and functional characteristics of turmeric-dyed natural textiles. An orthogonal array design was utilized to simultaneously optimize the turmeric dye extraction process and the subsequent textile dyeing conditions, thereby enabling comprehensive process enhancement. Optimal extraction efficiency was attained under a material-to-liquor ratio of 1:8, employing 80% ethanol as a solvent at 80 °C for 140 min. The ideal dyeing conditions for cotton fabrics were pH 8.0, 100 min duration, and 70 °C temperature.

The dyed fabrics exhibited greater color intensity with the natural mordants used, especially pomegranate peel, compared to metallic mordants. With regard to UV protection, natural mordants generally performed equally or better than metallic mordants. Dyed cotton fabric treated with chitosan as a mordant achieved the highest UPF value of 84.64.

The turmeric-dyed samples exhibited antibacterial efficacy in inhibiting the growth of microorganisms (*S. aureus* and *E. coli*), which was enhanced by mordants, especially pomegranate peel. Although natural mordants sometimes improved the antioxidant activity of dyed samples to grades comparable to those obtained with metallic mordants, FeSO_4_-treated fabrics displayed the strongest antioxidant activity (78.48% for cotton). Fabrics subjected to both mordanting and dyeing processes exhibited significantly enhanced color fastness properties with moderate to excellent ratings, except for color change resistance to washing.

Overall, these findings demonstrate that turmeric-dyed natural fabrics mordanted with natural mordants represent a promising, eco-friendly approach to enhancing dyeing properties and functionality in textiles. This study presents multiple avenues for further research. Future work will exploit the synergistic effects of low-concentration natural and mineral mordants to enhance fabric functionalities and reduce the environmental burden of the mordanting process. Mineral-based natural mordants contain trace metals with lower but non-negligible ecological risks compared with conventional metallic mordants, which requires further in-depth exploration of their environmental impacts. Curcumin fixation on cotton fabrics is dominated by mordant anchoring and chemical bonding, and this mechanism can be further verified via EDS and other energy spectrum analyses. Furthermore, systematic quantitative research on curcumin adsorption onto cotton fabrics will be implemented from four aspects: adsorption model, adsorption kinetics, maximum adsorption capacity, and adsorption mechanism, so as to supplement and improve the theoretical basis of natural dyeing technology. Building upon the findings presented herein, additional efforts should be directed toward identifying and systematically evaluating alternative natural mordants compatible with a wide range of textile substrates and natural fibers, thereby broadening the practical application scope of eco-friendly natural dyes.

## Figures and Tables

**Table 1:** Orthogonal design analysis of dye liquor absorbance.

Items	A	B	C	D	Absorption (λmax = 422 nm)
1	A_1_	B_1_	C_1_	D_1_	0.848
2	A_1_	B_2_	C_2_	D_2_	0.719
3	A_1_	B_3_	C_3_	D_3_	1.880
4	A_2_	B_1_	C_2_	D_3_	0.578
5	A_2_	B_2_	C_3_	D_1_	0.899
6	A_2_	B_3_	C_1_	D_2_	0.547
7	A_3_	B_1_	C_3_	D_2_	0.466
8	A_3_	B_2_	C_1_	D_3_	0.358
9	A_3_	B_3_	C_2_	D_1_	0.546
*K*_1_	3.447	1.892	1.753	2.293	
*K*_2_	2.024	1.976	1.843	1.732	
*K*_3_	1.370	2.973	3.245	2.816	
*k*_1_	1.149	0.631	0.584	0.764	
*k*_2_	0.675	0.659	0.614	0.577	
*k*_3_	0.457	0.991	1.082	0.939	
*R*	0.692	0.360	0.497	0.361	
Factor ranking	A > C > D > B				
Optimal factors	A_1_C_3_D_3_B_3_				

A, material-to-liquor ratio; B, duration of extraction; C, temperature of extraction; D, ethanol concentration. (kij = (Σ(h)ij)/3 (h = absorption values; i = 1, 2, 3; j = A, B, C, D); Rj = max(kij) − min(kij) (for the same factor j) (i = 1, 2, 3; j = A, B, C, D)).

**Table 2:** The color strength (*K/S*) values and corresponding range analysis results for dyed cotton fabrics obtained through the orthogonal experimental design.

No.	E	F	G	*K/S*
1	E_1_	F_1_	G_1_	4.49
2	E_1_	F_2_	G_2_	4.40
3	E_1_	F_3_	G_3_	4.38
4	E_2_	F_1_	G_2_	3.99
5	E_2_	F_2_	G_3_	2.75
6	E_2_	F_3_	G_1_	4.34
7	E_3_	F_1_	G_3_	2.41
8	E_3_	F_2_	G_1_	3.65
9	E_3_	F_3_	G_2_	4.29
*K*_1_	13.27	10.89	12.48	
*K*_2_	11.08	10.80	12.68	
*K*_3_	10.35	13.01	9.54	
*k*_1_	4.42	3.63	4.16	
*k*_2_	3.69	3.60	4.23	
*k*_3_	3.45	4.34	3.18	
*R*	0.97	0.74	1.05	
Factor ranking	G > E > F			
Optimal factors	G_2_E_1_F_3_			

E, temperature for dyeing; F, duration of dyeing; G, pH. (kij = (Σ(h)ij)/3 (h = absorption values; i = 1, 2, 3; j = E, F, G); Rj = max(kij) − min(kij) (for the same factor j) (i = 1, 2, 3; j = E, F, G)).

**Table 3:** Color strength and colorimetric parameters of optimally turmeric-dyed cotton fabrics following treatment with different mordants.

Treatment	L	a	b	∆*E*	*K/S*	Sample
None	48.42	21.95	32.06	45.66	7.63	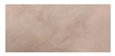
MgCl_2_	46.76	24.18	32.00	47.88	8.44	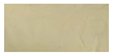
KAl(SO_4_)_2_	47.28	23.47	34.61	48.58	9.64	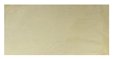
KCl	48.95	22.65	37.74	48.96	9.73	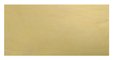
Rare earth	48.40	22.58	44.08	53.42	14.40	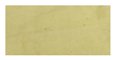
Pomegranate peel	52.71	19.93	50.53	54.81	16.26	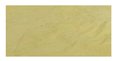
*C. speciosa* fruits	49.45	21.48	43.79	52.14	13.55	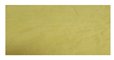
Mineral salts	43.01	26.38	38.42	55.00	15.79	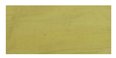
Tartaric acid	44.54	24.22	38.46	52.98	16.14	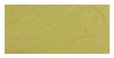
ZnSO_4_	47.48	23.54	37.71	50.29	10.60	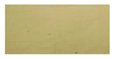
CaCl_2_	44.97	25.42	33.91	50.73	10.70	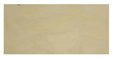
FeSO_4_	46.02	23.90	36.49	50.70	11.46	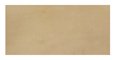
SnCl_2_	51.99	21.08	42.25	49.44	10.11	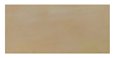
AgNO_3_	43.97	25.28	32.31	50.55	11.40	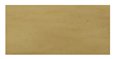
Plant ash	50.17	21.43	43.30	51.36	12.79	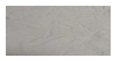
Chitosan	45.86	24.45	40.89	53.70	15.41	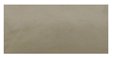
Gum rosin	45.37	25.13	40.04	53.81	14.54	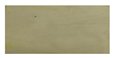
Soybean seed	50.55	21.07	46.20	53.08	12.28	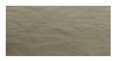
CuSO_4_	45.76	24.02	33.79	49.43	10.81	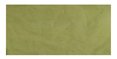

Note: L, lightness value; a, red–green chromatic coordinate; b, yellow–blue chromatic coordinate; ΔE, total color difference calculated against the untreated undyed cotton reference sample; *K/S*, color strength (color depth) calculated by the Kubelka–Munk equation. “None” represents the control group dyed without any mordant.

**Table 4:** Transmittance in cotton fabrics dyed with an optimized turmeric extract.

Mordant	Transmittance (%)	
	UV-A (315–400 nm)	UV-B (280–315 nm)
Undyed	10.77	9.45
Chitosan	1.16	1.04
Soybean seed	1.40	1.33
CuSO_4_	2.10	1.92
Pomegranate peel	2.80	2.58
FeSO_4_	2.85	2.72
Plant ash	2.94	2.80
AgNO_3_	3.09	3.10
Mineral salts	2.88	3.00
Rare earth	3.19	3.22
None	3.31	3.40
MgCl_2_	3.50	3.46
KCl	3.50	3.47
CaCl_2_	3.58	3.48
Gum rosin	3.80	3.55
KAl(SO_4_)_2_	3.76	3.71
SnCl_2_	4.13	3.64
*C. speciosa* fruits	3.89	3.72
ZnSO_4_	3.92	4.04
Tartaric acid	4.75	4.84

**Table 5:** Ultraviolet protection in cotton fabrics dyed with an optimized turmeric extract.

Mordant	UV Protection (%)	
	UV-A (315–400 nm)	UV-B (280–315 nm)
Undyed	89.23	90.55
Chitosan	98.84	98.96
Soybean seed	98.60	98.67
CuSO_4_	97.90	98.08
Pomegranate peel	97.20	97.42
FeSO_4_	97.15	97.28
Plant ash	97.06	97.20
AgNO_3_	96.91	96.90
Mineral salts	97.12	97.00
Rare earth	96.81	96.78
None	96.69	96.60
MgCl_2_	96.50	96.54
KCl	96.50	96.53
CaCl_2_	96.42	96.52
Gum rosin	96.20	96.45
KAl(SO_4_)_2_	96.24	96.29
SnCl_2_	95.87	96.36
*C. speciosa* fruits	96.11	96.28
ZnSO_4_	96.08	95.96
Tartaric acid	95.25	95.16

**Table 6:** Comparison of antibacterial inhibition zones for turmeric-dyed cotton fabrics in the presence and absence of mordant.

Treatment	ZOI (Diameter, mm)	
	*S. aureus*	*E. coli*
None	3.6 ± 0.3	3.3 ± 0.2
MgCl_2_	8.5 ± 0.3	4.9 ± 0.5
KAl(SO_4_)_2_	7.6 ± 0.4	7.5 ± 0.3
KCl	8.0 ± 0.3	5.5 ± 0.4
Rare earth	8.7 ± 0.4	5.8 ± 0.4
Pomegranate peel	10.2 ± 0.4	8.4 ± 0.3
*C. speciosa* fruits	8.1 ± 0.4	5.4 ± 0.3
Mineral salts	6.2 ± 0.2	5.2 ± 0.5
Tartaric acid	7.7 ± 0.4	5.1 ± 0.4
ZnSO_4_	7.8 ± 0.4	4.6 ± 0.4
CaCl_2_	8.3 ± 0.5	5.6 ± 0.5
FeSO_4_	9.4 ± 0.5	4.3 ± 0.3
SnCl_2_	9.6 ± 0.4	6.7 ± 0.4
AgNO_3_	8.9 ± 0.5	6.2 ± 0.3
Plant ash	7.9 ± 0.4	4.7 ± 0.3
Chitosan	5.8 ± 0.3	4.4 ± 0.3
Gum rosin	6.5 ± 0.3	4.5 ± 0.4
Soybean seed	5.2 ± 0.5	4.1 ± 0.3
CuSO_4_	9.8 ± 0.5	7.9 ± 0.3

**Table 7:** Impact of different mordants on color fastness of turmeric-dyed cotton samples.

Mordant	Washing Fastness			Rubbing Fastness		Perspiration Fastness			
						Acidic		Alkaline	
	CC	CS	∆*E*	Dry	Wet	CC	CS	CC	CS
None	1	3–4	7.25	4	3–4	1–2	4–5	1–2	3–4
Soybean seed	2	5	5.32	5	4–5	3	5	3	5
Rare earth	2	5	4.61	4–5	5	3	5	3	4–5
Chitosan	2	5	2.98	4–5	5	3	5	3	4–5
Pomegranate peel	2	5	3.49	4–5	4–5	3–4	5	4	4–5
Gum rosin	2	5	4.35	4–5	5	4–5	5	4	4–5
*C. speciosa* fruits	2	5	3.72	5	5	4–5	5	3–4	5
Plant ash	2	5	6.13	4–5	5	3	5	3	5
Mineral salts	2	5	4.19	5	4–5	3	4–5	3	4–5
Tartaric acid	2	5	2.96	4–5	4–5	4	4–5	3–4	4
KAl(SO_4_)_2_	2	5	3.54	4–5	5	3	5	3	4–5
SnCl_2_	2	5	4.47	5	4	3–4	4–5	4–5	5
CuSO_4_	2–3	4	1.76	4–5	4	3	4–5	3–4	4–5
FeSO_4_	3	4–5	1.03	4–5	4–5	3	5	3–4	5
ZnSO_4_	2	4	3.52	4–5	4–5	3	4–5	3–4	4–5
KCl	2	5	2.23	5	4–5	3	5	3–4	4–5
MgCl_2_	2	5	4.07	5	4–5	3	5	3–4	5
CaCl_2_	2	5	3.62	5	4–5	3	4–5	3–4	4–5
AgNO_3_	2	5	2.73	5	4–5	3	5	4	5

CC, color change; CS, color staining; 1, very poor; 2, poor; 3, moderate; 4, good; 5, excellent.

**Table 8:** Information of natural mordants.

Type of Natural Mordant	Mordant	Exact Source	Preparation Method & Yield	Main Active Ingredients	Optimal pH
Plant-based	Pomegranate Peel	Dried exocarp of pomegranate	Hot-water extraction 80–95 °C, liquor ratio 1:10–1:15; tannin yield 18–24%	Hydrolyzable tannins, gallic acid, ellagic acid, polyphenols	3.8–4.5
	*C. speciosa* fruits	Dried fruit of *C. speciosa*	Hot-water extraction 70–85 °C, liquor ratio 1:12; yield 12–17%	Malic acid, citric acid, tannins, flavonoids	3.5–4.2
	Soybean seed	Mature seeds of soybean	Warm-water soaking 40–50 °C, liquor ratio 1:20; yield 8–13%	Glycinin, plant proteins, peptides	6.0–6.8
	Plant ash	Ash from burning plant straw	Water soaking, liquor ratio 1:8; yield 15–21%	Potassium carbonate, potassium bicarbonate	9.0–10.0
	Tartaric acid	Fermentation by-product of grape	Acidification and recrystallization of crude tartar; yield 55–65%	L-(+)-tartaric acid (hydroxy organic acid)	3.2–4.0
Biopolymer-based	Chitosan	Deacetylated shrimp/crab shells	Hot alkali deacetylation; dissolved in acetic acid for dye bath; yield 70–80%	Poly-β-1,4-D-glucosamine (aminopolysaccharide)	5.0–6.0
Mineral-based	Mineral salts	Alunite ore	Crushing, water leaching, recrystallization; yield 40–52%	Potassium aluminum sulfate KAl(SO_4_)_2_·12H_2_O	4.2–5.0
	Rare earth	Separated and purified from light rare earth ores	Acid leaching, solvent extraction, crystallization; lanthanum recovery 65–75%	La^3+^ lanthanum ions	4.5–5.2
Resin-based	Gum rosin	Resin from Masson pine and other pines	Distillation to remove turpentine, yielding crude rosin; yield 65–72%	Resin acids (abietic acid, pimaric acid)	6.5–7.3

**Table 9:** Factors and levels for the orthogonal experimental design in turmeric root extraction.

Level	Factors			
	A. Material-to-Liquor Ratio	B. Time (min)	C. Temperature (°C)	D. Ethanol Concentration (%)
1	1:8	100	60	60
2	1:10	120	70	70
3	1:12	140	80	80

**Table 10:** Orthogonal experimental design: factor levels for optimizing turmeric dyeing on cotton fabrics.

Level	Factor		
	E. Temperature (°C)	F. Time (min)	G. pH
1	70	80	7.0
2	80	90	8.0
3	90	100	9.0

## Data Availability

The original contributions presented in this study are included in the article/Supplementary Materials. Further inquiries can be directed to the corresponding authors.

## Supplementary Materials

The following supporting information can be downloaded at: https://www.mdpi.com/article/10.3390/molecules31183278/s1.
